# Modelling the impact of the long-term use of insecticide-treated bed nets on *Anopheles* mosquito biting time

**DOI:** 10.1186/s12936-017-2014-6

**Published:** 2017-09-15

**Authors:** Claudia P. Ferreira, Silas P. Lyra, Franciane Azevedo, David Greenhalgh, Eduardo Massad

**Affiliations:** 1 Departamento de Bioestatística, IBB, UNESP, Botucatu, SP 18618-689 Brazil; 2 Faculdade de Computação e Engenharia Elétrica, UNIFESSPA, Marabá, PA 68507-590 Brazil; 3 Department of Mathematics and Statistics, University of Strathclyde, Glasgow, G1 1XH Scotland; 4 School of Medicine, University of São Paulo, São Paulo, SP 01246-903 Brazil

**Keywords:** Agent based model, Mosquito plasticity, Sensitivity analysis, Fitness

## Abstract

**Background:**

Evidence of changing in biting and resting behaviour of the main malaria vectors has been mounting up in recent years as a result of selective pressure by the widespread and long-term use of insecticide-treated bed nets (ITNs), and indoor residual spraying. The impact of resistance behaviour on malaria intervention efficacy has important implications for the epidemiology and malaria control programmes. In this context, a theoretical framework is presented to understand the mechanisms determining the evolution of feeding behaviour under the pressure of use of ITNs.

**Methods:**

An agent-based stochastic model simulates the impact of insecticide-treated bed nets on mosquito fitness by reducing the biting rates, as well as increasing mortality rates. The model also incorporates a heritability function that provides the necessary genetic plasticity upon which natural selection would act to maximize the fitness under the pressure of the control strategy.

**Results:**

The asymptotic equilibrium distribution of mosquito population versus biting time is shown for several daily uses of ITNs, and the expected disruptive selection on this mosquito trait is observed in the simulations. The relative fitness of strains that bite at much earlier time with respect to the wild strains, when a threshold of about 50% of ITNs coverage highlights the hypothesis of a behaviour selection. A sensitivity analysis has shown that the top three parameters that play a dominant role on the mosquito fitness are the proportion of individuals using bed nets and its effectiveness, the impact of bed nets on mosquito oviposition, and the mosquito genetic plasticity related to changing in biting time.

**Conclusion:**

By taking the evolutionary aspect into account, the model was able to show that the long-term use of ITNs, although representing an undisputed success in reducing malaria incidence and mortality in many affected areas, is not free of undesirable side effects. From the evolutionary point of view of the parasite virulence, it should be expected that plasmodium parasites would be under pressure to reduce their virulence. This speculative hypothesis can eventually be demonstrated in the medium to long-term use of ITNs.

## Background

Malaria continues to be responsible for between 16 and 24% of total child deaths in sub-Saharan Africa, which represents between 100,000 and 500,000 deaths per year [1]. The upper limit of these figures means 1 child less than 5 years of age dying every minute [2]. These figures, however, are 25% lower than those observed before the successful intervention by entities such as the Global Fund to Fight AIDS, Tuberculosis and Malaria, and the Bill and Melinda Gates Foundation [3]. These initiatives are responsible for the majority of global funding for malaria intervention programmes and have distributed hundreds of millions of insecticide-treated bed nets (ITNs) since the beginning of the last decade [1]. The impact of these measures on malaria mortality has been throughly reported [2]. In some African countries such as Ghana [5] under-five mortality among children who sleep under treated bed nets is about 18.8% lower than among children who do not sleep under treated bed nets.

The current strategy of malaria control, therefore, is mainly relying on indoor residual insecticide spraying and the distribution of ITNs. The combination of these two strategies has proved to be very effective against the main malaria vectors in Africa, *Anopheles gambiae* and *Anopheles funestus* [2], both mosquitoes which bite and rest indoors at night, which is the period that people are sleeping [4, 5]. These mosquitoes show intense anthopophilic and endophilic behaviour, which make them very competent vectors of malaria [4]. The night-biting preference of these mosquitoes explains the efficacy of ITNs in Africa due to the double effects of ITNs in reducing the biting rates—they provide a physical barrier against bites [6]—and repelling or killing mosquitoes (or both)—the insecticide that impregnates the nets has a powerful knock-down and killing effect [7]. This, together with the programmes of indoor spraying of insecticide, have been the most important factors in reducing malaria incidence and mortality observed in the last decade [8].

Mosquitoes, however, are not a passive and static species and have been adopting survival and reproductive strategies, for example increasing their resistance against insecticides, shifting their biting habits to earlier hours and changing to outdoor biting behaviour [2]. It should, therefore, have been expected that endophilic malaria vectors, such as *A. gambiae* and *A. funestus* would gradually change their feeding and resting behaviour as a reaction against the current strategies of indoor spreading of insecticides and the distribution of ITNs. In fact, evidence of changes in biting and resting behaviour of malaria vectors, have been mounting up in recent years [9–13]. The presence of outdoor-activity, early-biting malaria vectors have been reported in many countries in Africa, such as Benin [14], Tanzania [15], Kenya [16–18], Nigeria [19], Uganda [20], Gambia [21], Ghana [22] and Rwanda [23], amongst others.

In this paper a stochastic model is proposed to simulate the evolution of biting behaviour change of mosquitoes due to the use of ITNs, based on the theoretically expected reduction in biting rates and knock-down, killing effects, or both, provided by the physical barrier and insecticide effects of ITNs [24, 25]. The model uses *ad hoc* functions to reproduce the impact of ITNs on the fitness of mosquitoes by reducing the biting rates and increasing mortality rates. In addition, the model incorporates a heritability function that provides the necessary genetic plasticity to the population of mosquitoes, upon which natural selection would act to maximize the fitness under the pressure of the control strategy [13, 26].

The model is not intended to provide a public health tool to help decision-makers in guiding their malaria control strategies but rather present a theoretical framework on which experiments can be carried out to better understand the mechanisms determining the evolution of feeding behaviour under the pressure of the use of ITNs. Moreover, the model does not deal with insecticide resistance but rather concentrates on the effects of ITNs on the feeding and resting behaviour of malaria mosquitoes. This paper is organized as follows: After this brief introduction, the details of the model are presented in the methods section. The results of the numerical simulations are presented in a specific section on results and the final comments and conclusions presented in “Discussion” section.

## Methods

An individual based simulation model was used to construct a population of *n* female mosquitoes that survive during both phases of their lifecycle, immature and adult, taking blood meals to produce viable female offspring to form the next generation. Non-overlapping generations were considered, and a time step was defined as the update of the *n* individuals in the population. At each time step the mosquito population achieved its carrying capacity *C*. The carrying capacity was assumed to be a monotonic decreasing function of *k*, since carrying capacity is determined by food availability, according to the equation

1 $$\begin{aligned} C=Me^{-k^2}, \end{aligned}$$

where *k* is the product of the maximum proportion of individuals using bed nets and effectiveness of bed nets, and *M* represents the carrying capacity of the environment for mosquitoes when bed nets are not used. As $k \in [0,1]$, *C* varies between *M* / *e* and *M* which means that when $k=1$ bed nets can reduce the mosquito population by up to 63%. In [25] a reduction of 64–92% in mosquito biting when ITNs are used is reported. Notice that to achieve a 20% reduction in *C*, $k \approx 0.47$ is needed. At the beginning of a simulation each individual mosquito $i, \ i \in 1, 2, \ldots n$, is assigned a biting time parameter $h_i\in [0,24[$ hours according to a Gaussian distribution corresponding to the number of hours after 12:00 p.m. at which the mosquito bites, so for example a mosquito assigned a number 5.00 will always bite at 17:00 p.m. This parameter is genetically determined and corresponds to the time of day or night at which the mosquito bites.

For each individual mosquito the impact of the use of bed nets by humans is represented by the parameter $z_i$ according to

2 $$\begin{aligned} \quad z_i = k e^{\frac{-(h_i - h_m)^2}{2\sigma _1^2}} \end{aligned}$$

where $h_m$ represents the time in hours measured from 12:00 p.m. where the bed net usage is greatest and $\sigma _1$ is a constant time representing the variability of the bed net usage over a night about its maximum.

For mosquito *i* the parameter $\mu _i \in [0,1]$ represents the probability that that particular mosquito dies. $\mu _i$ is given by the equation

3 $$\begin{aligned} \mu _i = \mu _N+\frac{z_i}{z_i+\alpha _1}, \end{aligned}$$

where $\mu _N$ represents a natural probability of death, and $\alpha _1 > 0$ is an arbitrary constant. As $\alpha _1$ increases, the impact of the use of bed nets on the mosquito mortality probability decreases.

The oviposition (birth) probability $\phi _i$ is given by

4 $$\begin{aligned} \phi _i = \max \Bigl (\epsilon e^{\frac{-(h_i - h_m)^2}{2\sigma _2^2}}-\alpha _2 z_i,0 \Bigr ). \end{aligned}$$

In the absence of bed nets $z_i=0$, $\phi _i$ is highest for a mosquito that bites at time $h_m$ because most people are asleep, so the mosquito will get a better blood meal. Again $\sigma _2$ is a constant time representing the variability over a night of the number of available hosts about its maximum value, $\alpha _2$ measures the impact of bed net use on oviposition probability, and $\epsilon$ is the maximum value of this probability.

Other functions can be used to simulate the impact of ITNs on the mosquito population, the ones proposed here were inspired by field experiments that relate host availability and mosquito captures inside and outside houses during the daytime [27, 28]. Observe that bed net impact on the carrying capacity is supposed to be multiplicative as the available blood meal is reduced by a fraction of the initial one when bed nets are not used ($k=0$), and additive in the case of mortality (or oviposition) rate, since natural and induced mortality are independent events. Furthermore, the effect of bed nets on mosquito mortality and oviposition (by feeding inhibition) is not the same [7, 25]. For example, the risk difference obtained comparing mosquito mortality risk and blood feeding risk when ITNs or untreated bed nets (UTNs) are used can be −0.66 to −0.27 for blood feeding and 0.74 to 0.39 for mosquito mortality; the variation in both is explained by the degree of resistance of the mosquitoes [25].

When the simulation is updated, first of all the individual mosquito survival probabilities are applied. For this, a mosquito is chosen, say with biting time $h_i$, at random and ask if it will survive comparing a randomly generated number from a Uniform distribution with its survival probability $\mu _i$. Then, the second step is, if it survives, it can produce a daughter with probability $\phi _i$. Again, a randomly generated number obtained from a Uniform distribution is compared with $\phi _i$ to decide if the mosquito will produce a daughter. This process is repeated until the total mosquito population size given by the carrying capacity is reached. Overall, one mosquito can produce more than one offspring depending on its mortality and oviposition probabilities. At the start of the next simulation step, that corresponds to a new mosquito population generation, each daughter mosquito is assumed to have reached full adult maturity.

**Fig. 1:**
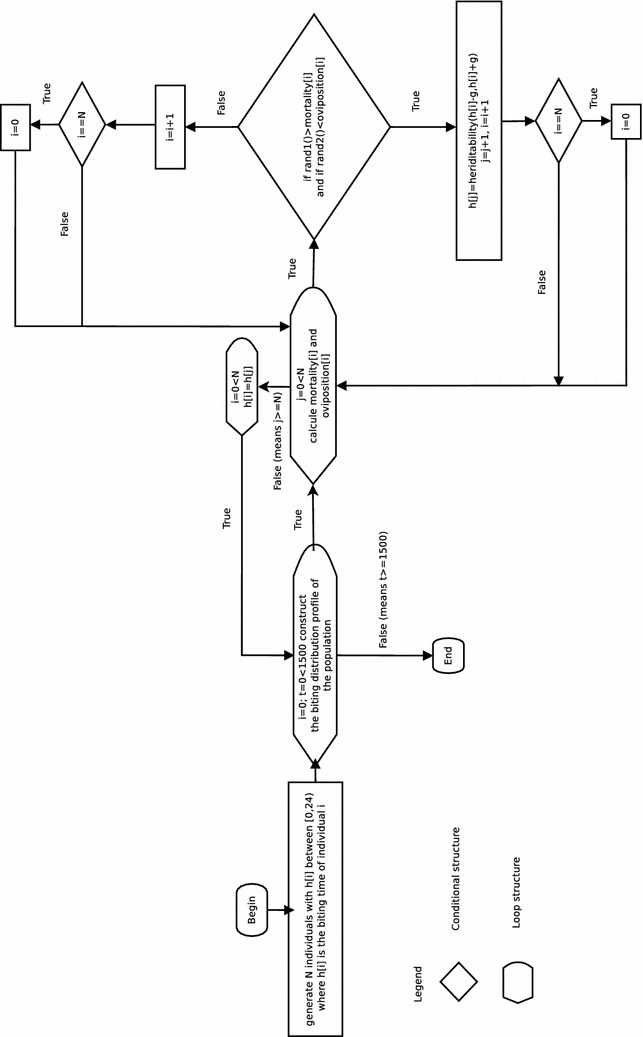
Algorithm of the agent-based model.This algorithm represents the main one developed for the agent based model and was implemented in the C language. All the results presented in the manuscript are mean values of 200 simulations of it. Specific subroutines were developed to obtain the results discussed and presented in the figures of the manuscript

When a mosquito assigned a biting parameter $h_i$ reproduces, her offspring is assigned a biting rate parameter which follows a Uniform distribution in

5 $$\begin{aligned}{}[h_i-g,h_i+g], \end{aligned}$$

where *g* is a model parameter. The larger *g* is the greater the possible difference between the biting time of a mother mosquito and her daughter. This corresponds to a genetic plasticity so the offspring have the potential to adapt to the use of bed nets to maximize their survival and reproductive potential [13]. Observe that the biting time of a daughter depends on the biting time of her mother. Figure 1 shows the algorithm proposed.

The curve of mosquito population distribution versus biting time can be characterized by several quantities such as the number of maxima and minima, the position of the maxima and minima, the population size at these points, the variance of each peak, and the area under this curve. In particular, the positions of the maxima and minima, and the variance of each peak are measured by taking the first and second derivative of the distribution of the mosquito population biting time.

Finally, the relative fitness of the individuals in the population that bite at a specific time $h_i$ is evaluated as

6 $$\begin{aligned} F_i= \frac{N_i}{C}, \end{aligned}$$

where $N_i$ is the number of individuals biting at time $h_i,$ and *C* is the carrying capacity.

A sensitivity analysis is performed to assess the strength of the relationship between each one of the model parameters and the population fitness. For this, the space of the input values is first sampled using a Latin Hypercube Sampling (LHS) with $\mu _N \sim Unif(0.05,0.15),$ $\sigma _1\sim Unif(10,20)\;({\text{h}}),$ $\sigma _2 \sim Unif(250,350)\;({\text{h}}),$ $\alpha _1 \sim Unif(10,20),$ $\alpha _2 \sim Unif(0,0.1),$ $\epsilon \sim Unif(0.4,0.8)$ $g \sim Unif(0.1,1)$ (hours), and $k\sim Unif(0,0.8)$ where *Unif* means a Uniform probability distribution. LHS is a stratified sampling without replacement technique that ensures that the entire range for each parameter is explored. The Monte Carlo simulation is done by drawing a set of *L* independent parameters from the Uniform distribution and evaluating $F=\sum F_i$. The sum is done in a chosen interval of biting time. For this, $L=2000$ and the period of 13–14 for the interval of biting time are used, i.e., the fitness of the individuals that bite around 1:30 a.m. is evaluated. Assuming that the relation between output and input is linear, a regression model can be used to assess the sensitivity of *F* to each model parameter. The standardized regression coefficients (SRCs) can be used for sensitivity analysis provided that the model coefficient of determination $R^2$ is 0.7 or higher [29, 30]. Some parameter values and their ranges for the sensitivity analysis are inspired by the literature (for example $\mu _N$, $\epsilon$, $h_m$, and $\sigma _1$) and others are reasonably chosen (e.g. $\sigma _2>>\sigma _1$) [31, 32].

## Results

In all simulations $\sigma _1^2=15\;{\text{h}},$ $\sigma _2^2=300\;{\text{h}},$ $\alpha _1=15,$ $\alpha _2=0.05,$ $\epsilon =0.6,$ $\mu _N=0.1,$ $h_m=14.5$ hours (which means 2:30 a.m.), and $M=10,000$ were used. The results are the mean values obtained from 200 simulations. Each simulation is run 1500 times to guarantee that a stable equilibrium distribution of the mosquito population biting time profile is achieved.

**Fig. 2:**
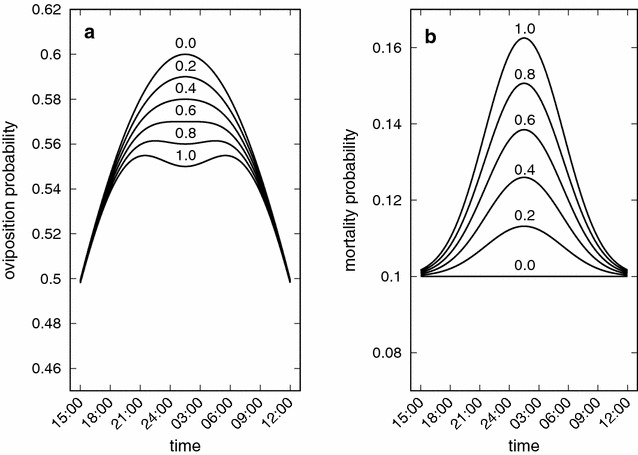
Oviposition and mortality of mosquitoes. Oviposition and mortality probabilities versus mosquito biting time for different proportions of individuals using bed nets, *k*. In **a** *k* increases from top to bottom and in** b** it increases from bottom to top. Each curve was obtained for the specific value of *k* adressed in the figure. The others parameters are $\sigma _1=15\;{\text{h}},$ $\sigma _2=300\;{\text{h}},$ $\epsilon =0.6, \alpha _1=15,$ $\alpha _2=0.05$ and $\mu _N=0.1$

Figure 2 shows the oviposition and mortality probabilities versus mosquito biting time given by Eqs. (3) and (4). In general, the use of bed nets increases the mosquito mortality probability and decreases the mosquito oviposition probability, affecting mostly the individuals that bite around 2:30 a.m. when bed net use is at its maximum. In [31] the authors argue that in most countries more than 75% of anopheline bites would be prevented by bed nets because biting rhythm and the times when most people go to bed coincide (from 22:00 to 05:00 h in rural areas). The data shown are from mosquito captures up to the year 2004.

**Fig. 3:**
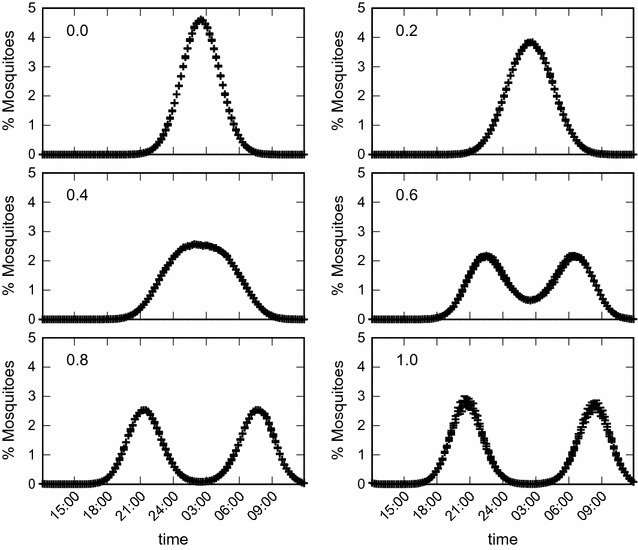
Population biting time profile of mosquitoes. Stable equilibrium distribution of the mosquito population biting time profile for different proportions of individuals using bed nets. The initial condition is given by a Gaussian function *N*(14.5, 4). The results are the mean values of 200 simulations with $g=0.3\;{\text{h}},$ $\sigma _1=15\;{\text{h}},$ $\sigma _2=300\;{\text{h}},$ $\epsilon =0.6,$ $\alpha _1=15,$ $\alpha _2=0.05$ and $\mu _N=0.1$.

**Fig. 4:**
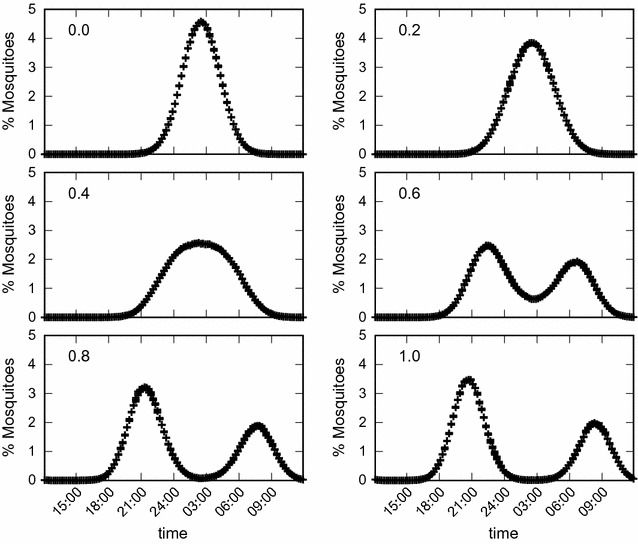
Population biting time profile of mosquitoes. Stable equilibrium distribution of the mosquito population biting time profile for different proportions of individuals using bed nets. The initial condition is given by a Gaussian function *N*(13.5, 4). The results are the mean values of 200 simulations with $g=0.3\;{\text{h}},$ $\sigma _1=15\;{\text{h}},$ $\sigma _2=300\;{\text{h}},$ $\epsilon =0.6,$ $\alpha _1=15,$ $\alpha _2=0.05$ and $\mu _N=0.1$.

Figures 3 and 4 show the long-term asymptotic equilibrium mosquito population distribution as a function of the biting time. The results are obtained using $g=0.3\;{\text{h}}.$ It can be seen in Figs. 3 and 4 that without the use of bed nets this distribution has its maximum at $h_m$ hours, and mosquitoes bite preferentially from 24:00 p.m until 4:00 a.m. As the use of bed nets increases, initially the maximum value of this distribution decreases and its variance increases. Then at $k=0.6$ a split of the mosquito biting time distribution into two peaks, one centered at 22:00 p.m. and the other at 06:00 a.m., is observed. These two figures are generated using different initial conditions: in Fig. 3 a Gaussian function centered at 14.5, *N*(14.5, 4) is used, and in Fig. 4 the Gaussian function is centered at 13.5, *N*(13.5, 4). As a result, a symmetric and an asymmetric distribution for the stable equilibrium mosquito distribution population profile were obtained, respectively. Overall, the appearence of the two peaks represents an adaption of this population due to the selection pressure of the bed net use which selects individual mosquitoes which are able to have their blood meals at those times of day when humans are awake and not using bed nets.

**Fig. 5:**
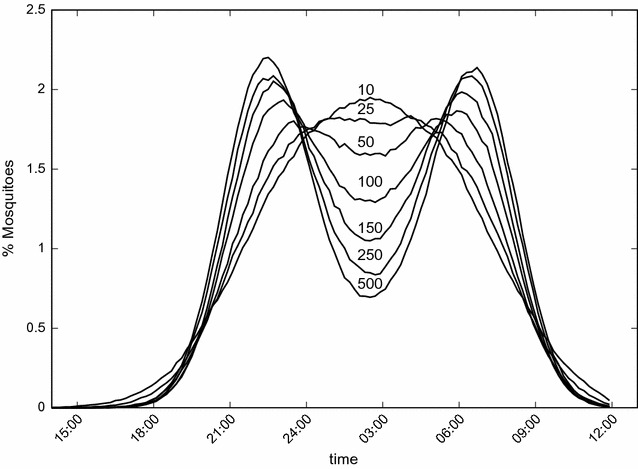
Evolution of the mosquito population biting time profile. Each curve was obtained at a specific time adressed in the figure. The results are the mean values of 200 simulations with $\sigma _1=15\;{\text{h}},$ $\sigma _2=300\;{\text{h}},$ $\epsilon =0.6,$ $\alpha _1=15,$ $\alpha _2=0.05$ and $\mu _N=0.1$.

Figure 5 shows the evolution in time of the mosquito biting time distribution using $g=0.3$ and $k=0.6$. It can be seen that the stable equilibrium distribution is achieved at $t \approx 500$. Starting from a Gaussian distribution of biting time the mosquito population evolves to achieve a specific profile of biting time distribution that results from the selection pressure of bed net use that selects individuals more adapted to this new environment.

**Fig. 6:**
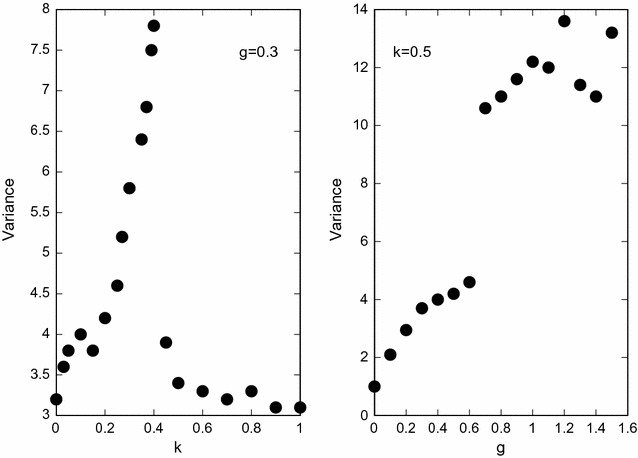
Population biting time variance of mosquitoes. On the left mosquito population distribution variance versus the proportion of individuals using bed nets, and on the right mosquito population distribution variance versus the amount of genetic plasticity. The results are the mean values of 200 simulations with $\sigma _1=15\;{\text{h}},$ $\sigma _2=300\;{\text{h}},$ $\epsilon =0.6,$ $\alpha _1=15,$ $\alpha _2=0.05$ and $\mu _N=0.1$.

Figure 6 shows how the mosquito biting time distribution depends on the proportion of individuals using bed nets, *k*, and on the plasticity parameter, *g*. Fixing *k* and increasing *g*, a transition that marks the appearance of a unique peak, characterized by a large variance can be seen. The same occurs when *g* is fixed and *k* is increased. The sudden diminution of the variance is a signal of the appearance of two peaks in the mosquito population distribution.

**Fig. 7:**
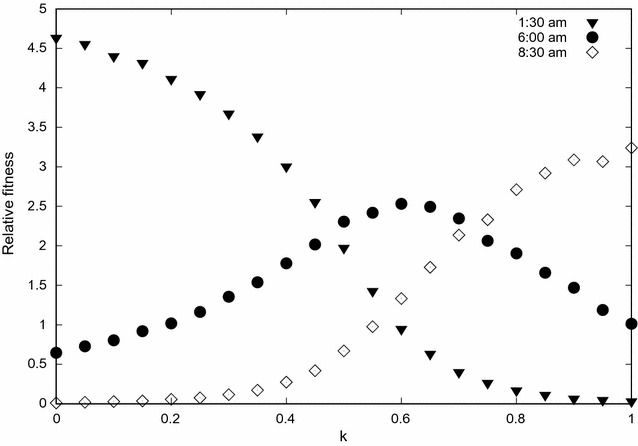
Relative fitness of mosquito population. Relative fitness versus the proportion of individuals using bed nets. The results are the mean values of 200 simulations with $g=0.3\;{\text{h}},$ $\sigma _1=15\;{\text{h}},$ $\sigma _2=300\;{\text{h}},$ $\epsilon =0.6, \alpha _1=15,$ $\alpha _2=0.05,$ and $\mu _N=0.1.$ Each curve represents a population with a specific biting time.

Figure 7 shows the relative fitness of the population as a function of bed net use for three different biting times. At low levels of coverage, the relative fitness of the resident population is biggest as a result of the abundance of blood hosts. As the use of bed nets increases, the protective barrier around sleeping humans increases mosquito mortality and makes it difficult for them to access a blood meal promoting the emergence of a “mutant” population characterized by a distinct biting time. Because the stable equilibrium distribution of the mosquito population biting time profile depends on *k*, this new “mutant population” can coexist with another one or displace it depending on the shape of the oviposition and mortality probability. This behaviour change of the population characterizes a response to the new environmental conditions imposed by the intense use of ITNs.

**Fig. 8:**
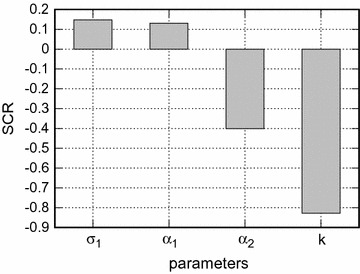
Sensitivity analysis. The sensitivity of the parameters $\sigma _1, \alpha _1, \alpha _2, k$ to the wild population fitness.

**Fig. 9:**
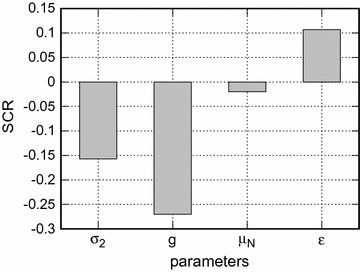
Sensitivity analysis. The sensitivity of the parameters $\sigma _2, g, \mu _N, \epsilon$ to the wild population fitness

Figures 8 and 9 show the standard regression coefficients (SRCs) obtained from the sensitivity analysis. The model coefficient of determination is $R^2=0.84$. The sensitivity based on the SRC can capture 100% of the variation in *F*. The parameter *k* accounts for 69% ($0.83^2$) of this variation. An increase in *k* promotes a decrease in *F*. Another parameter that plays an important role on the population fitness is $\alpha _2$. It accounts for $16\%$ of the variation in *F*. The other parameters together explain just $15\%$ of the variation in the population fitness. Overall, increases in $\sigma _2$, *g*, $\alpha _2$, *k*, and $\mu _N$ decrease *F*, while increases in $\sigma _1$, $\alpha _1$ and $\epsilon$ increase *F*. Finally, the order of importance of the parameters of the model on the fitness is $k, \alpha _2, g, \sigma _2, \sigma _1, \alpha _1, \epsilon$ and $\mu _N$.

## Discussion

In this paper an agent-based simulation model is proposed to test the hypothesis that due to long-term use of insecticide impregnated bed nets to control malaria transmission, change in the biting time behaviour of mosquitoes is likely to be observed. This reasoning is based on the assumption that both the physical barrier provided by the nets and the insecticide effect would reduce the biting rate and increase the repellent effect or mortality rate of the mosquitoes. This combined effect, which is the ultimate aim of the use of insecticide-impregnated bed nets in malaria control, is expected to force the disruptive or directional evolution of biting time, shifting the wild range of nocturnal habits of *Anopheles* mosquitoes towards daytime. The model reproduces the theoretical evolutionary effects that would be expected due to the selective pressure represented by the long-term use of ITNs. By including functions that reproduce the impact of ITNs on the carrying capacity of mosquitoes (Eq. 1), the individual impact on the biting time of each mosquito as a function of the use of ITNs (Eq. 2), the mortality rate (Eq. 3), and the oviposition rate (Eq. 4), the expected disruptive selection on the biting time of mosquitoes is observed in the simulations. Figure 2 shows how the oviposition rate decreases and the mortality rate increases as a function of the proportion of people using ITNs. The shift in biting time away from the wild average towards both earlier and later times is clearly observed in Figs. 3 and 4. In addition, there is clear increase in the relative fitness of strains that bite at much earlier time with respect to the wild strains, when a threshold of about 50% of use of ITNs is reached, above which the mutant strains overwhelm the wild strain (Fig. 7).

The impact of ITNs on behaviour of mosquitoes has been addressed previously [13, 25, 26] but the current model, by taking the evolutionary aspect into account [27], was able to demonstrate that the long-term use of ITNs, although representing an undisputed success in reducing malaria incidence and mortality in many affected areas, is not free of undesirable side effects.

If, on the one hand, the long-term use of ITNs may eventually induce important changes in the biting behaviour of mosquitoes, shifting their average biting time from nocturnal to daylight, remarkably reducing the efficacy of ITNs, on the other hand, from the evolutionary point of view of the parasite virulence [33, 34], it should be expected that *Plasmodium* parasites would be pressed to reduce their virulence. This reasoning is based on the so-called “Ewald hypothesis” [33, 34], according to which it should be in the evolutionary interest of the pathogen to keep a high virulence when circulating among an unprotected host population but reduce it when some control measure changes the natural cycle of the disease. In an unprotected population, *Plasmodium* strains that keep the host very ill, if possible laying in bed and unreactive against the mosquito bites, should cause a serious disease. As the use of ITNs presses the mosquitoes to change their biting habits towards daytime, it would be in the evolutionary interest of those parasites to cause a less severe disease. This however, is only a speculative hypothesis that could possibly eventually be demonstrated in the medium to long-term of use of ITNs.

Finally, one aspect not considered in this study, but which may have important repercussions on the efficacy of ITNs in malaria control, is the possibility of evolution of resistance against the insecticide by the mosquitoes. This effect has been observed in areas with particularly high pyrethroid resistance [35].

As mentioned in the introduction, the current study is not intended to provide a public health tool to help decision-makers in guiding their malaria control strategies but rather represents only a theoretical framework on which hypotheses can be tested and experiments can be carried out to better understand the mechanisms determining the evolution of feeding behaviour under the pressure of the use of ITNs.
